# Supplementary material for: Visualizing the internal structure of the charge-density-wave state in CeSbTe
Source: Nat Commun. 2025 Mar 28;16:3053. doi: 10.1038/s41467-025-58417-x (PMC11953432; doi:10.1038/s41467-025-58417-x)
Supplement: Supplementary file 1 — Supplementary Information [file 41467_2025_58417_MOESM1_ESM.pdf]

Supplementary Information

Supplementary Note 1: Tight-binding model, intraorbital and interorbital nesting

We elaborate on the tight-binding model presented in Fig. 1 of the main text. As shown in Supplementary Fig. 1a, we consider degenerate, half-filled $p_{x'}$ and $p_{y'}$ orbitals in the (x', y') frame of the square lattice and three distinct hopping terms: (1) t_σ, nearest-neighbour hopping between two $p_{x'}$ or two $p_{y'}$ orbitals with σ overlap; (2) t_π, nearest-neighbour hopping between two $p_{x'}$ or two $p_{y'}$ orbitals with π overlap; (3) t', next-nearest-neighbour, interorbital hopping. In Fig. 1b of the main text, a schematic Fermi surface with only the t_σ term is drawn. Here, in Supplementary Fig. 1b, we consider realistic values for all three terms: t_σ = 1.65 eV, t_π = 0.5 eV, and t' = 0.08 eV. The effect of the t_π term is to relieve the 1D propagation of $p_{x'}$ and $p_{y'}$ electrons, allowing them to hop also along the y' and x' directions, respectively. The quasi-1D $p_{x'}$ and $p_{y'}$ sheets acquire some warping along the $k_{y'}$ and $k_{x'}$ directions, respectively. The effect of the t' term is to create hybridization gaps at the crossing points between $p_{x'}$ and $p_{y'}$ sheets along the Γ-M' line.

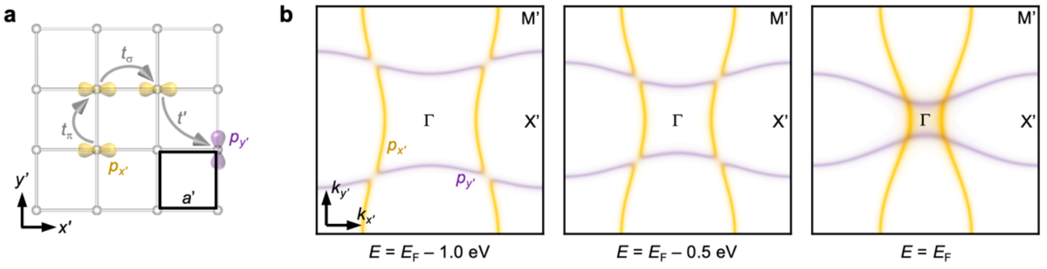

Supplementary Fig. 1: Tight-binding model of *p*-electron square lattice. a, Schematic diagram with orbitals and hopping parameters labeled. The black box encloses the unit cell. **b**, Computed Fermi surface at various different energies, decomposed into $p_{x'}$ and $p_{y'}$ orbital contributions (t_σ = 1.65 eV, t_π = 0.5 eV, t' = 0.08 eV, and E_F = 1 eV). A broadening of 0.075 eV was applied in the plot of these Fermi surfaces.

To model the Sb 5p bands in CeSbTe, we need to account for the fact that the unit cell of CeSbTe contains two Sb atoms; i.e., it is a $\sqrt{2} \times \sqrt{2}$ enlargement of the unit cell of an isolated Sb square lattice. Supplementary Fig. 2a shows the doubled unit cell in the crystallographic frame (x, y) of CeSbTe, which is a 45° rotation of the local frame of the Sb square lattice (x', y'). Supplementary Fig. 2b shows the corresponding folded Brillouin zone. We retain the orbital basis ($p_{x'}$, $p_{y'}$) defined on the original square lattice, as well as the hopping terms t_σ, t_π, and t'. We build the following 4×4 Hamiltonian in the basis ($p_{x'1}$, $p_{y'1}$, $p_{x'2}$, $p_{y'2}$), where 1 and 2 refer to the two Sb atoms in the unit cell:

$$H = \begin{pmatrix} H_{11} & H_{12} & H_{13} & H_{14} \\ H_{21} & H_{22} & H_{23} & H_{24} \\ H_{31} & H_{32} & H_{33} & H_{34} \\ H_{41} & H_{42} & H_{43} & H_{44} \end{pmatrix}, \qquad (1)$$

$$H_{12} = 2t'\left(\cos k_y a_0 - \cos k_x a_0\right) = H_{21} = H_{34} = H_{43},$$

$$H_{13} = 2t_\sigma \cos((k_x + k_y)a_0/2) - 2t_\pi \cos((k_x - k_y)a_0/2) = H_{31},$$

$$H_{24} = -2t_\pi \cos((k_x + k_y)a_0/2) + 2t_\sigma \cos((k_x - k_y)a_0/2) = H_{42},$$

and all other matrix elements are zero.

The resulting band structure is shown in Supplementary Fig. 2c. The hopping parameters, t_σ = 1.65 eV, t_π = 0.5 eV, and t' = 0.08 eV, as well as the Fermi energy E_F = 1.0 eV, were chosen to best approximate the Sb 5p bands in the band structure of $CeSb_{0.63}Te_{1.37}$ computed by density functional theory (DFT; Supplementary Fig. 2d). There are linear band crossings at the Fermi energy along the Γ-X and Γ-M paths, which, in the case of $CeSb_{0.63}Te_{1.37}$, are shifted by roughly 1 eV below E_F due to electron doping from Te substitutions, and partially gapped by hybridization to Te bands and spin-orbit coupling. There are also degenerate points at X, which remain ungapped in $CeSb_{0.63}Te_{1.37}$ due to protection by nonsymmorphic symmetry (*1*). Supplementary Fig. 2e shows the Fermi surface at various energies. At E_F, the Fermi surface comprises a diamond-shaped pocket centered at Γ, made of $p_{x'}$ and $p_{y'}$ segments. Above E_F, the Fermi surface splits into two concentric diamond-shaped pockets. As marked by the wave vectors **q** (gray arrows), there are nested intraorbital $p_{x'} \rightarrow p_{x'}$ segments and nested intraorbital $p_{y'} \rightarrow p_{y'}$ segments, giving rise to a $p_{x'}$ density wave and a $p_{x'}$ density wave, whose relative spatial phase is then determined by additional local interactions.

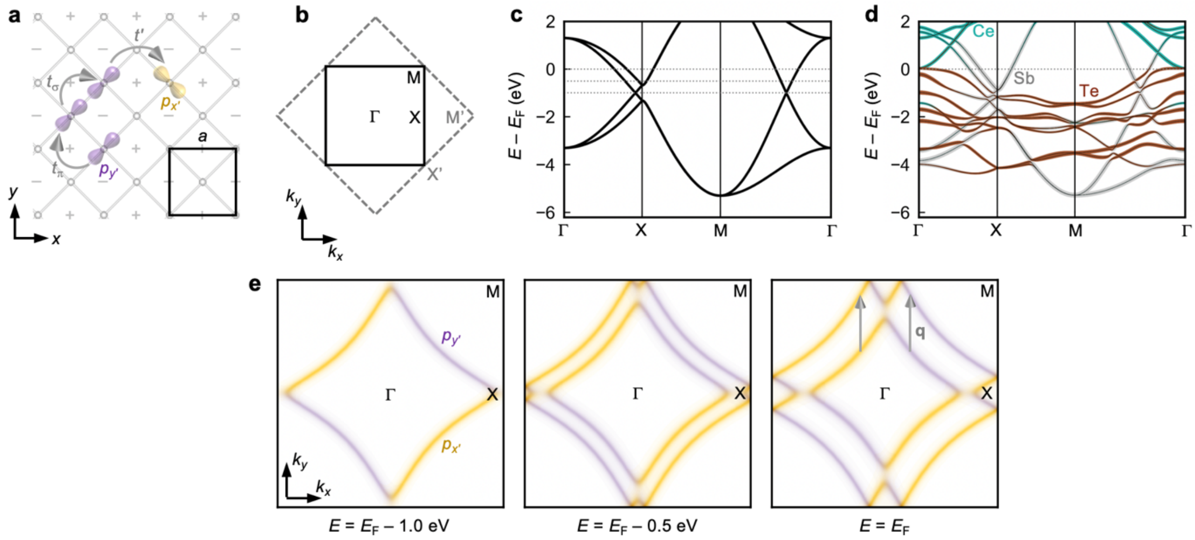

Supplementary Fig. 2: Tight-binding model of *p*-electron square lattice with enlarged two-atom unit cell. a, Schematic diagram with orbitals and hopping parameters labeled. The + and – signs refer to Ce atoms staggered above and below the Sb square lattice in CeSbTe, giving rise to nonsymmorphic symmetry. The black box labels the enlarged unit cell. **b**, Original (dotted line) and folded (solid line) Brillouin zones. **c**, Computed band structure (t_σ = 1.65 eV, t_π = 0.5 eV, t' = 0.08 eV, and E_F = 1 eV). **d**, Comparison with DFT-computed band structure of $CeSb_{0.63}Te_{1.37}$ (reproduced from Fig. 1h of the main text). **e**, Computed Fermi surface at various different energies, decomposed into $p_{x'}$ and $p_{y'}$ orbital contributions. The gray arrows represent intraorbital nesting conditions, $p_{x'} \rightarrow p_{x'}$ and $p_{y'} \rightarrow p_{y'}$. A broadening of 0.15 eV was applied in the plots of these Fermi surfaces.

In Supplementary Fig. 3, we present the same Fermi surfaces computed in Supplementary Fig. 2 based on Supplementary Eq. (1), but with a change in the orbital basis to (p_x, p_y), where $p_x = (p_{x'} + p_{y'})/\sqrt{2}$ and $p_y = (p_{x'} - p_{y'})/\sqrt{2}$. This new basis manifests an interorbital component to the nesting conditions. As shown in Supplementary Fig. 3b, the equivalent wave vector **b*** – **q**, where **b*** is a reciprocal lattice vector, connects $p_x \rightarrow p_y$ segments (longer gray arrow). As suggested by Wang *et al.*, these interorbital nesting conditions could lead to a density wave with mixed p_x and p_y orbital characters in $GdTe_3$ and $LnTe_3$; i.e., with finite orbital angular momentum (2). In the case of our 1×7 charge-density-wave (CDW) state, the nesting of these p_x and p_y orbitals, which are hybridized combinations of $p_{x'}$ and $p_{y'}$ orbitals, may play a role in fixing the relative phase of the $p_{x'}$ and $p_{y'}$ bond density waves. These p_x and p_y orbitals may also play a role in hybridizing with Ce $dx^2–y^2$ orbitals at +1.0 eV, as their lobes extend along the *a* and *b* directions and directly overlap with those of Ce $dx^2–y^2$.

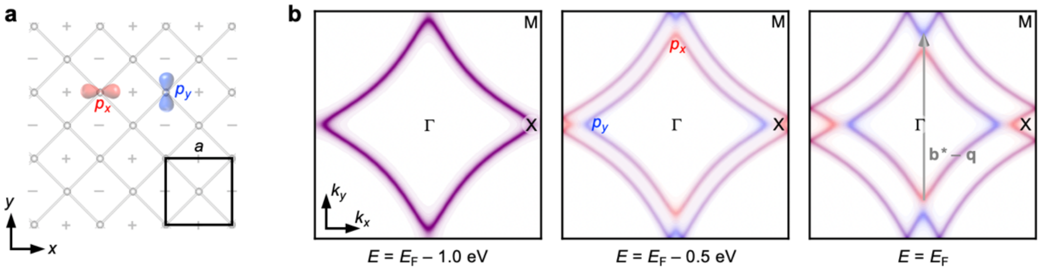

Supplementary Fig. 3: Change of orbital basis. a, Same tight-binding model as in Supplementary Fig. 2a, but in the basis of p_x and p_y orbitals defined relative to the (x, y) frame of CeSbTe, rather than the (x', y') frame of the isolated Sb square lattice. **b**, Computed Fermi surface at different energies, decomposed into p_x and p_y orbital contributions (t_σ = 1.65 eV, t_π = 0.5 eV, t' = 0.08 eV, and E_F = 1 eV). The gray arrow represents interorbital nesting ($p_x \rightarrow p_y$). A broadening of 0.15 eV was applied in the plots of these Fermi surfaces.

Supplementary Note 2: Estimate of doping level

In the synthesis of CeSbTe, Te has a strong propensity to replace Sb and/or leave behind vacancies, leading to nonstoichiometric crystals with chemical formula Ce($Sb_{1-x}Te_x$)$Te_{1-\delta}$. Since we cleave between Te planes, we could directly visualize and count the Te vacancies to determine δ (see Supplementary Note 3). However, we could not image any atomic-sized features that could correspond to Te substitutions in subsurface Sb layer, which are the source of additional electrons. To estimate the actual doping level of our samples, we made use of the prior observation that within a wide range of Te substitutions, the CDW wave vector **q** grows linearly with increasing *x* (*3*). This dependence implies that **q** tracks the nesting wave vector of the Fermi surface, which increases linearly with energy due to the linear dispersion of the Dirac bands. By extracting **q** from our STM topographies, we could work backwards and estimate the effective doping level by computing the corresponding Fermi surface that would yield a nesting wave vector of **q**.

We performed DFT calculations of stoichiometric CeSbTe in its 1×1×1 tetragonal phase using experimental lattice parameters of $a = b = 4.409(1)$ Å and $c = 9.368(2)$ Å (*4*). Supplementary Fig. 4a shows the calculated band structure. We then computed the Fermi surfaces at various energies. At the Fermi energy E_F (Supplementary Fig. 4b), the Sb 5*p* bands form a diamond-shaped pocket, while the Te 5*p* bands form a hole pocket at Γ that is irrelevant to our discussion. At energies above E_F (Supplementary Fig. 4c), the Sb 5*p* bands split into two concentric diamond-shaped pockets. To determine the nesting wave vector that is parallel to k_y (red arrow in Supplementary Fig. 4c), which is the experimentally observed CDW direction, we computed the autocorrelation of the Fermi surface (Supplementary Fig. 4d), then searched for the maximum intensity along the k_y axis. We found that the nesting wave vector had a value of 1/7th of the reciprocal lattice vector b^* when the energy was 0.68 eV above E_F. To convert the energy into a doping level, we further assumed that the doping could be described by a rigid band shift. We also assumed that the doping originates from Te substitutions at the Sb site, and that each Te substitution donates one additional electron. We then determined that a rigid band shift of +0.68 eV corresponds to a doping level of $x = 0.39$.

Supplementary Fig. 4e presents a phase diagram of CeSbTe with the doping concentration *x* on the horizontal axis (inverted) and the CDW wave vector **q** on the vertical axis. The data points represented by the green squares and the dashed lines depicting phase boundaries are derived from Singha *et al.* (*5*). Below $x \sim 0.3$, the unit cell of Ce($Sb_{1-x}Te_x$)$Te_{1-\delta}$ is tetragonal and no CDW was detected by x-ray diffraction. For *x* between roughly 0.3 and 0.74, Ce($Sb_{1-x}Te_x$)$Te_{1-\delta}$ becomes orthorhombic with unidirectional CDW. The Fermi surface in this region comprises nested diamond-shaped pockets (inset of Supplementary Fig. 2e). Above $x \sim 0.74$, Ce($Sb_{1-x}Te_x$)$Te_{1-\delta}$ remains orthorhombic, but multi-*q* CDW states are stabilized. This evolution may reflect the change in Fermi surface topology, where the inner diamond-shaped pocket splits into four pockets. Our samples, with *q* values determined from scanning tunneling microscopy (STM) and *x* values estimated using DFT, lie within the orthorhombic, single-*q*

phase of $Ce(Sb_{1-x}Te_x)Te_{1-\delta}$ (yellow squares in Supplementary Fig. 4e). Furthermore, they lie on a straight line joining the green squares, which validates the assumptions that went into our estimates of x.

In the main text, we performed DFT calculations using the virtual crystal approximation for a doping concentration of $x = 0.37$, rather than $x = 0.39$, because there are x-ray data with refined lattice parameters only for a $x = 0.37$ sample (5).

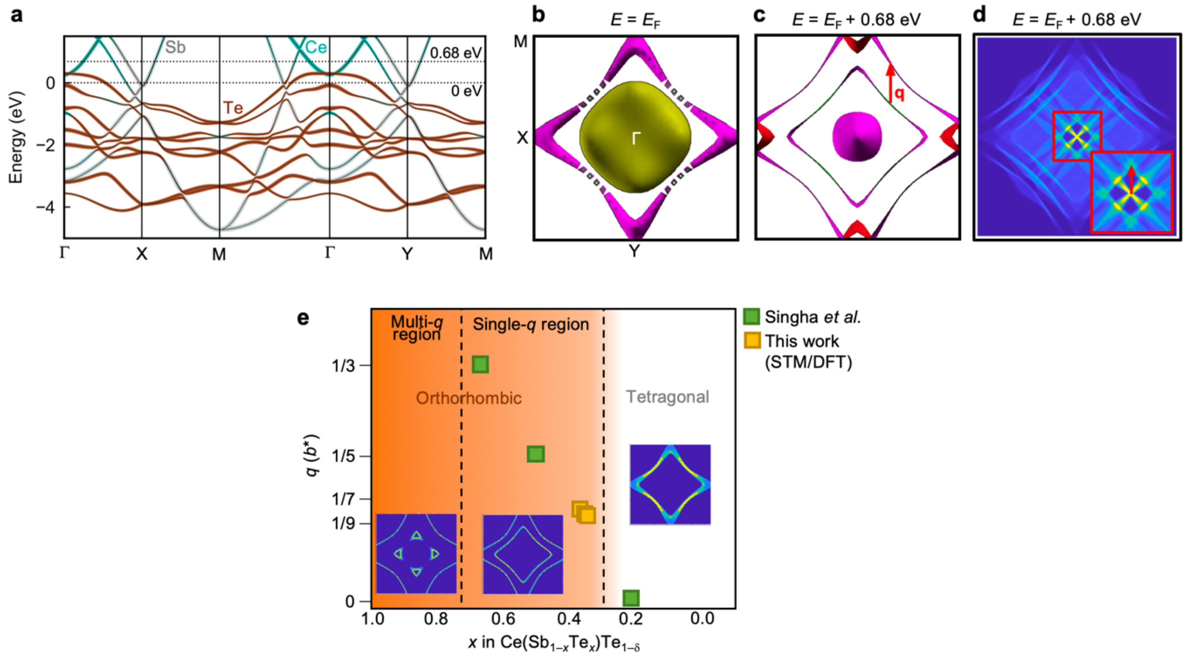

Supplementary Fig. 4: Estimate of doping level of samples. a, Band structure of stoichiometric CeSbTe in the 1×1×1 tetragonal phase, decomposed into atomic contributions (Ce = teal, Sb = gray, Te = brown). **b**, Calculated Fermi surface at the Fermi energy E_F. **c**, Calculated Fermi surface at +0.68 eV above E_F. **d**, Autocorrelation of **c**. The nesting wave vector of length $1/7 \times b^*$ is marked by the red arrows in **c** and in the inset of **d**. **e**, Phase diagram of $Ce(Sb_{1-x}Te_x)Te_{1-\delta}$, divided by dashed lines into three regions: tetragonal (white shading), orthorhombic with single-q CDW (orange shading), and orthorhombic with multi-q CDW (orange shading). The q values of the unidirectional CDW for samples with different stoichiometries are derived from Ref. (5) (green squares). The q values of the samples measured in this work, with stoichiometry estimated from a rigid band shift of DFT calculations, are shown in yellow squares. The insets show the DFT-computed Sb pockets in the three different regions of the phase diagram.

Supplementary Note 3: Te vacancy concentration and CDW wavelength

Our STM experiments have been carried out on three different Ce($Sb_{1-x}Te_x$)$Te_{1-\delta}$ samples. From numerous topographic images taken at negative sample-tip biases, in which we imaged a square Te lattice and CDW as a background amplitude modulation, we could compile statistics for the concentration of Te vacancies and CDW wavelengths. As shown in the histograms in Supplementary Fig. 5, our samples consistently had about 4% Te vacancies, or $\delta = 0.04$. The CDW wavelength is close to 7*a* in sample 1, from which the data in Figs. 2–6 of the main text were collected, and close to 8*a* in samples 2 and 3. We did not observe any clear correlation between the number of Te vacancies and the CDW wavelength (Supplementary Fig. 5c). As previously discussed, the CDW wavelength is more directly affected by the number (x) of Te substitutions in the Sb square lattice, which directly inject electrons into the "CDW-active" Sb layer.

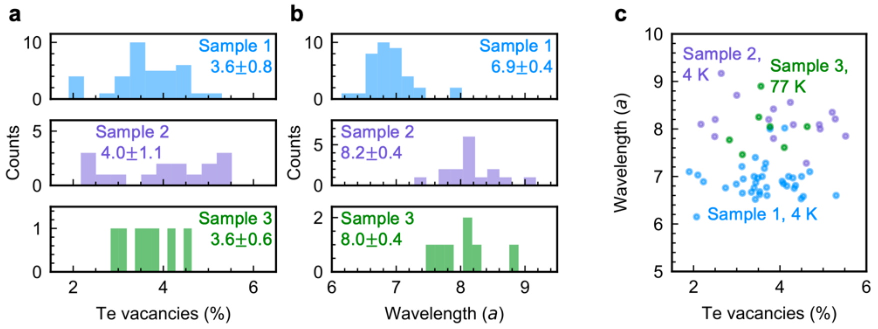

Supplementary Fig. 5: Sample statistics derived from negative-bias STM topographic images. a, Histogram of percentage of Te vacancies. **b**, Histogram of wavelength of CDW. **c**, Scatter plot of CDW wavelength vs. percentage of Te vacancies.

Supplementary Note 4: Magnetotransport

We performed electrical transport measurements of a single crystal $Ce(Sb_{1-x}Te_x)Te_{1-\delta}$ from the same synthesized batch as samples 1 and 2 measured with STM. Upon lowering of the temperature in zero field, the resistivity increases monotonically and a kink is observed at 2.6 K, as shown in Supplementary Fig. 6a, coinciding with an antiferromagnetic phase transition at this temperature. The overall weak insulating behavior and the additional upturn in resistivity below ~10 K have been discussed in literature as a possible consequence of a gap opening with the establishment of CDW order and Kondo scattering *(6)*. Magnetoresistance measurements at 0.35 K show a discontinuous jump at $\mu_0 H_c = \pm 0.27$ T (Supplementary Fig. 6b), coinciding with a ferromagnetic transition. Our data points lie close to the phase boundaries previously reported for CeSbTe (Supplementary Fig. 6c), and confirm that our STM measurements at 4.2 K and 0 T lie within the paramagnetic phase of CeSbTe.

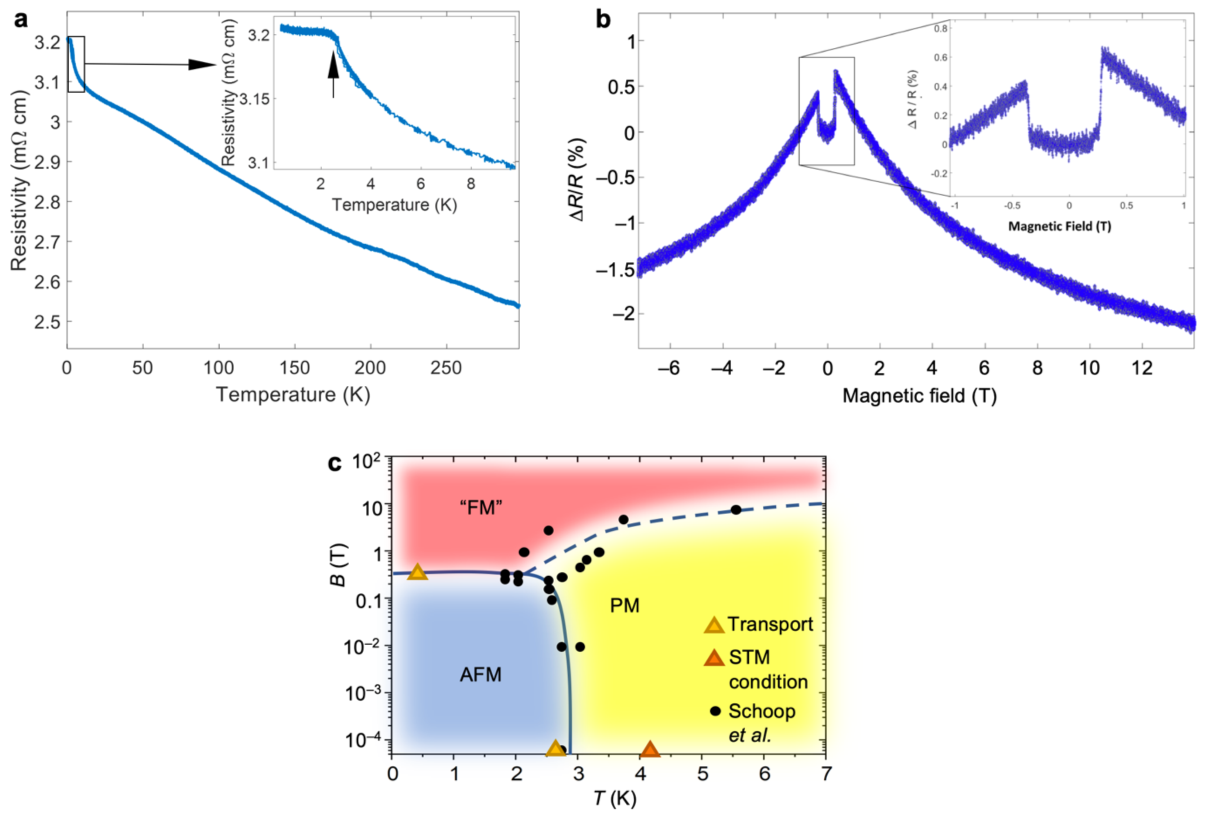

Supplementary Fig. 6: Magnetotransport characterization and phase diagram. a, Temperature-dependent electrical resistivity of a $Ce(Sb_{1-x}Te_x)Te_{1-\delta}$ sample from the same batch as those measured with STM. The arrow in the inset marks a kink at 2.6 K corresponding to an antiferromagnetic transition. **b**, Out-of-plane magnetoresistance measured at 0.35 K, showing a discontinuity at ±0.27 T corresponding to a ferromagnetic transition. **c**, Phase diagram of CeSbTe, showing paramagnetic (PM), antiferromagnetic (AFM) and ferromagnetic (FM) phases. The black circles are data points derived from Ref. *(4)*, and the solid and dashed lines are phase boundaries derived from those points. Additional data from our magnetotransport characterization in **a** and **b** are shown as yellow triangles. Our STM measurement conditions in the paramagnetic phase are represented by the orange triangle.

Supplementary Note 5: FT masks

Supplementary Fig. 7 shows the FT masks used to produce the filtered –0.5 and +1.0 V STM topographies shown in Figs. 4a and 5a of the main text.

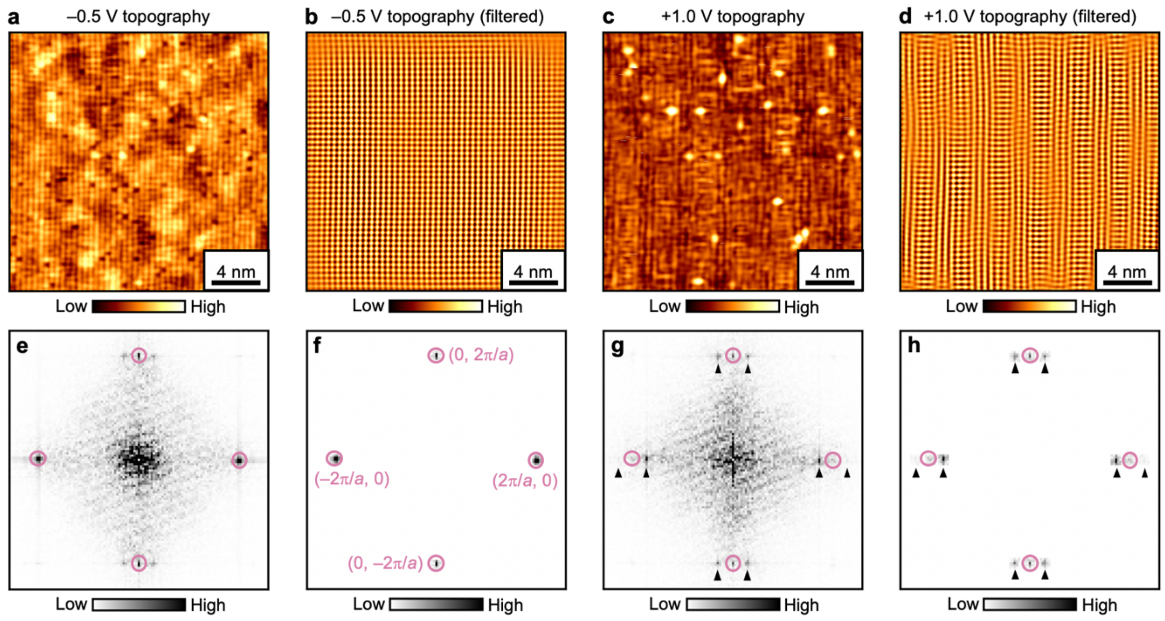

Supplementary Fig. 7: Topographic images and their FTs. a–**d**, Topographic images reproduced from Figs. 3a, 4a, 3b, and 5a of the main text, respectively. **e**–**h**, FTs corresponding to **a**–**d**. **f** and **h** are the masked FTs used to produce the filtered topographies of **b** and **d**.

Supplementary Note 6: Additional examples revealing CDW internal structure

Supplementary Fig. 8 shows –0.5 and +1.0 V topographies acquired over the same ~10×10 nm area using Multi Pass imaging mode. We repeat the same analyses applied in Figs. 4–6 of the main text to confirm the reproducibility of our results with a different data set.

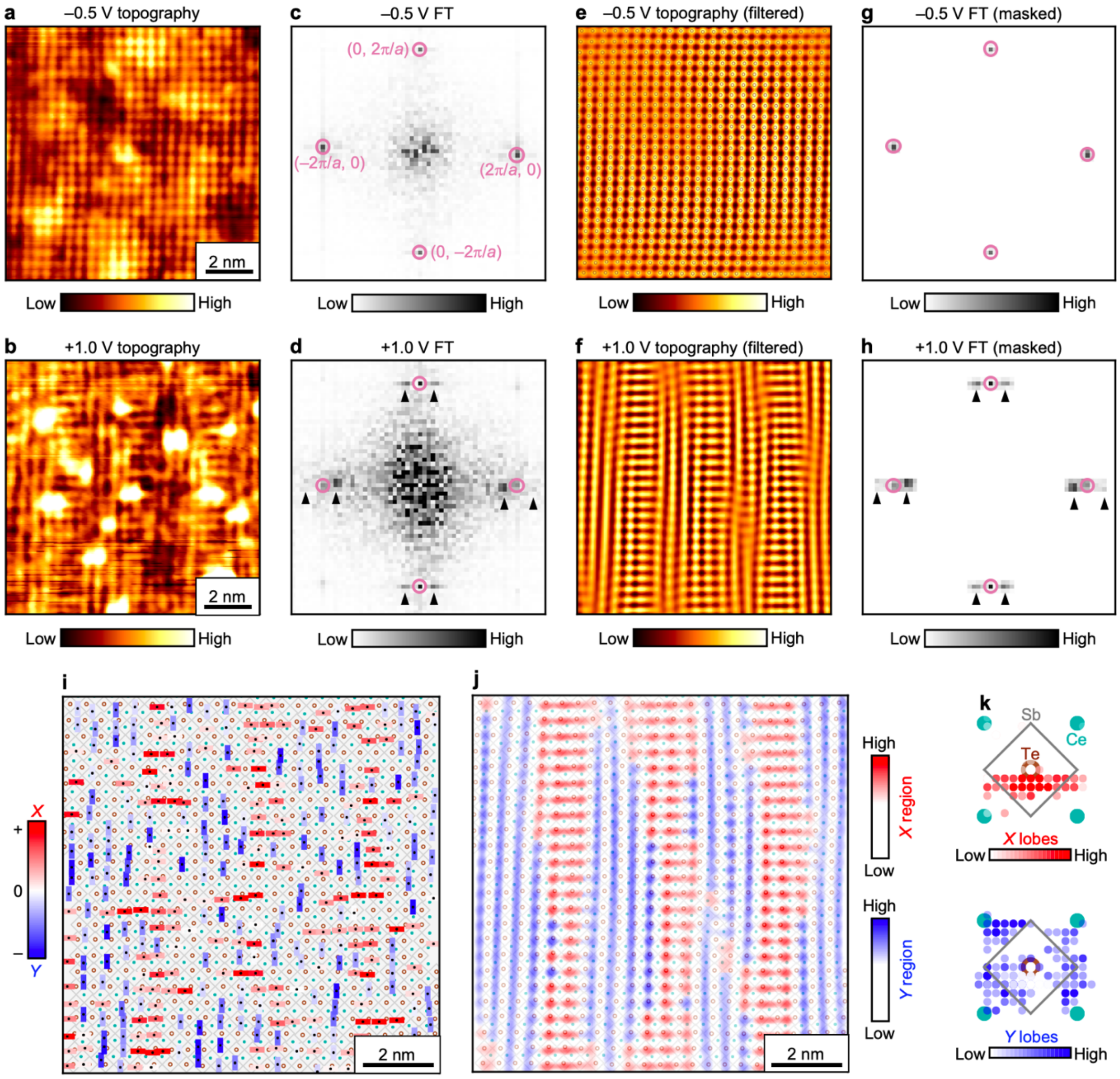

Supplementary Fig. 8: Additional topography (I). **a** and **b**, STM topographies of the same ~10×10 nm field of view imaged with –0.5 and +1.0 V biases, respectively. Setpoint current: 150 pA. **c** and **d**, FTs of **a** and **b**, respectively. The Bragg peaks and CDW satellite peaks are marked with pink circles and black triangles, respectively. **e** and **f**, Fourier-filtered images of **a** and **b**, respectively. The brown open circles mark the positions of the local maxima in **e**. **g** and **h**, Masked FTs used to generate **e** and **f**. **i**, Map of lobe anisotropy. Each black dot represents a lobe position, each rectangle indicates the orientation of the lobe along x or y, and the color of each rectangle indicates the degree of lobe anisotropy, as quantified by $\eta(x_i, y_i)$ in Eq. (1) of the main text. **j**, Filtered topography in **f** with red-blue binning applied to demarcate regions of predominant x (red) or y (blue) lobe anisotropy. **k**, Distribution of the positions of the x- and y-anisotropic lobes from **j** within a reference CeSbTe unit cell. The intensity of the red or blue colors is proportional to the number of anisotropic lobes at the given position.

Supplementary Figs. 9 and 10 show CDW domains over larger fields of view imaged at +0.5 V. The x- and y-anisotropic lobes are already visible at +0.5 V, though not as pronounced as at +1.0 V. These images demonstrate that the pattern of alternating x- and y-anisotropic lobes that composes the internal structure of the 1×7 CDW is coherent over tens and hundreds of nanometers.

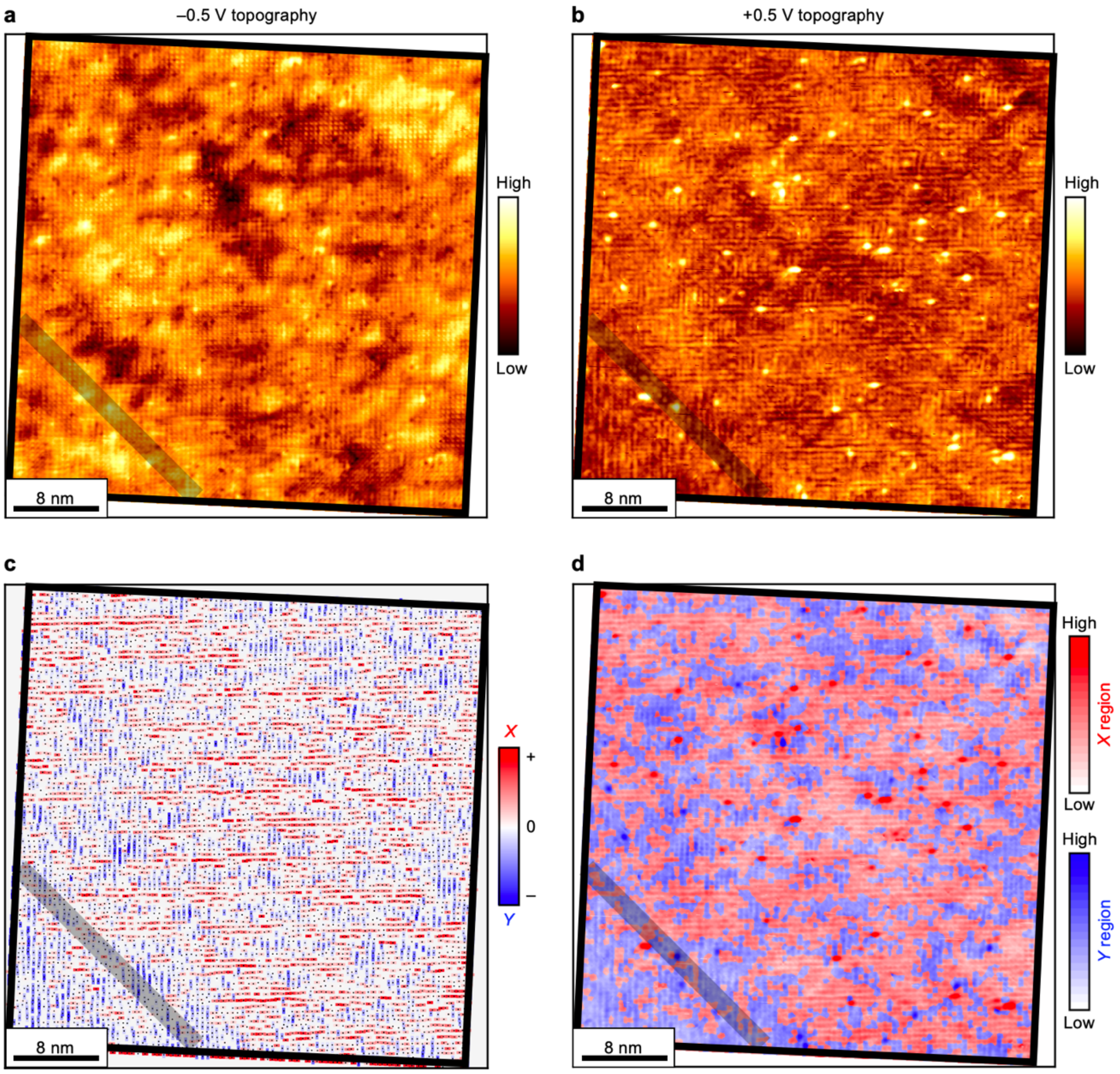

Supplementary Fig. 9: Additional topography (II). **a** and **b**, STM topographies of the same ~40×40 nm field of view imaged with –0.5 and +0.5 V biases, respectively. Setpoint current: 50 pA. The thick transparent line marks a domain boundary. **c**, Map of lobe anisotropy. Each black dot represents a lobe position, each rectangle indicates the orientation of the lobe along x or y, and the color of each rectangle indicates the degree of lobe anisotropy, as quantified by $\eta(x_i, y_i)$ in Eq. (1) of the main text. **d**, Topography in **b** with red-blue binning applied to demarcate regions of predominant x (red) or y (blue) anisotropy.

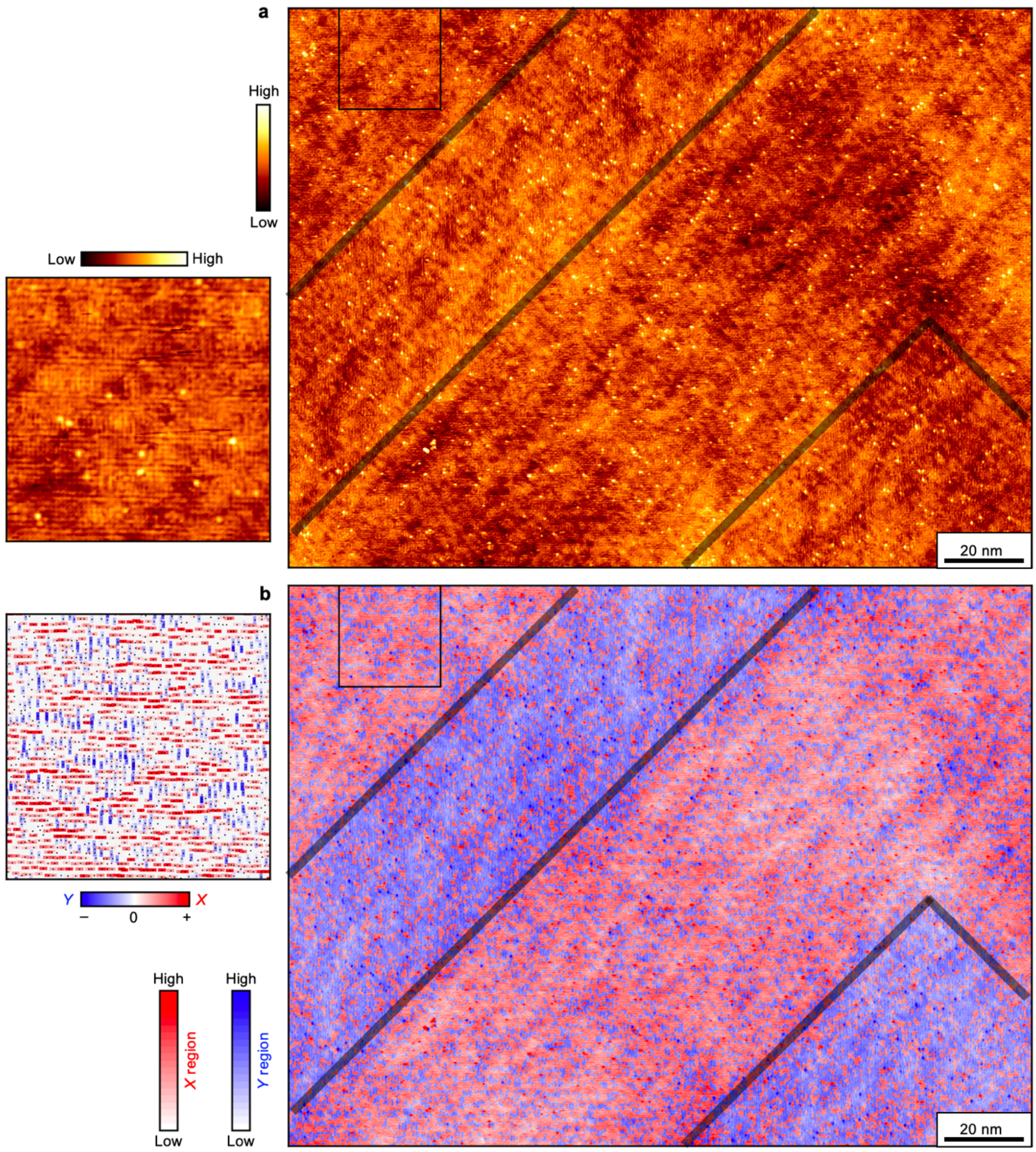

Supplementary Fig. 10: Additional topography (III). **a**, STM topography imaged at +0.5 V, showing a total of four CDW domains (boundaries marked by transparent lines). Setpoint current: 10 pA. The inset shows an enlarged view of the boxed region. **b**, Topography in **a** with red-blue binning applied to demarcate regions of predominant x (red) or y (blue) lobe anisotropy. Inset: Map of lobe anisotropy within the boxed region. Each black dot represents a bright lobe position, each rectangle indicates the orientation of the lobe along x or y, and the color of each rectangle indicates the degree of lobe anisotropy, as quantified by $\eta(x_i, y_i)$ in Eq. (1) of the main text. We note that the computation of x and y anisotropy in this image is somewhat skewed due to the low spatial resolution.

Supplementary Fig. 11 shows an example where the x and y anisotropy of the lobes of charge density are clear, but the modulation wave vector is not. The red and blue regions do not form clear stripes along either the x or y directions. Further work is needed to investigate the prevalence and

significance of these disordered regions, which resemble STM images seen in tetragonal $GdSb_{0.87}Te_{1.11}$ (7).

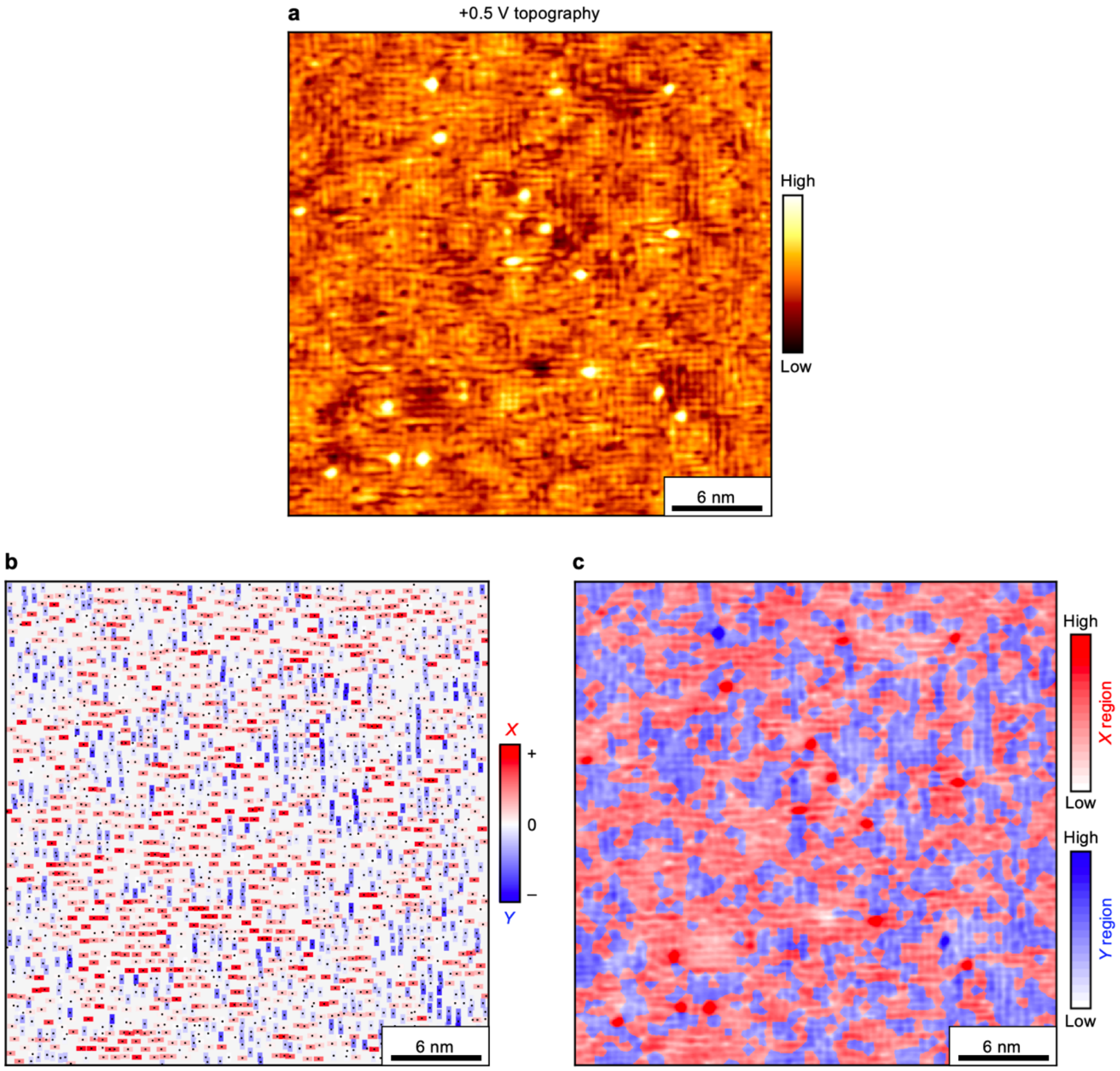

Supplementary Fig. 11: Additional topography (IV). **a**, STM topography imaged at +0.5 V. Setpoint current: 25 pA. **b**, Map of lobe anisotropy. Each black dot represents a lobe position, each rectangle indicates the orientation of the lobe along x or y, and the color of each rectangle indicates the degree of lobe anisotropy, as quantified by $\eta(x_i, y_i)$ in Eq. (1) of the main text. **c**, Topography in **a** with red-blue binning applied to demarcate regions of predominant x (red) or y (blue) anisotropy.

We consistently observed the same texture of alternating x/y-anisotropic lobes in various other channels beyond constant-current topographic imaging:

1. ***dI*/*dV* map** (Supplementary Fig. 12): Compared to bias-dependent topography $z(x, y, eV)$, which integrates states within an interval $[0, eV]$, the tunneling conductance $g(x, y, eV) = dI/dV(x, y, eV)$ probes states only near eV. We observed x/y-anisotropic lobes starting around +100 mV.

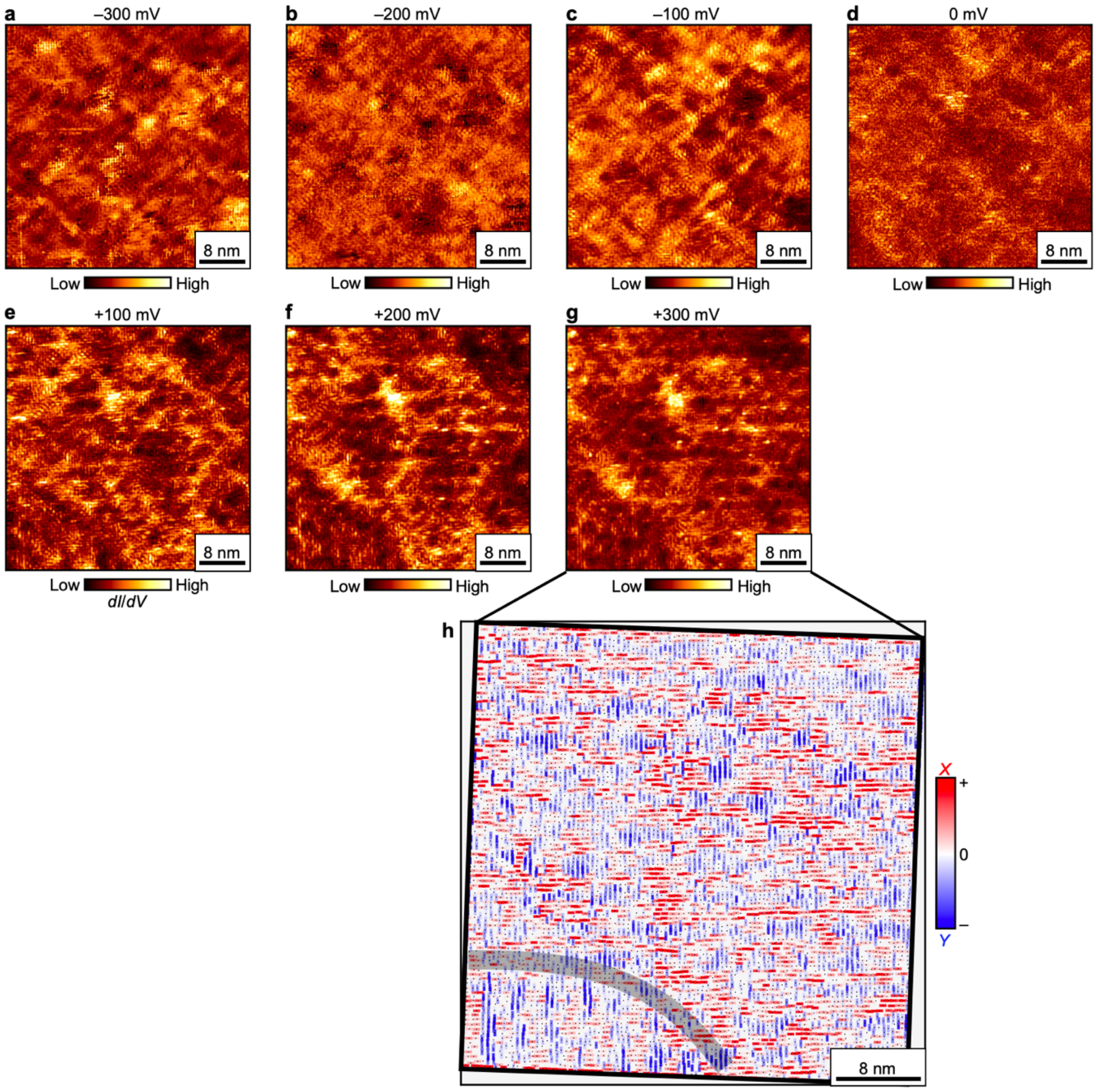

Supplementary Fig. 12: Tunneling conductance map. a–**g**, Selected energy slices of a 3D spectroscopic map, $g(x, y, eV)$. Setup conditions: –300 mV, 45 pA. Lock-in excitation: 28 mV. **h**, Map of lobe anisotropy extracted from **g**, the +300 mV layer. A 2° rotation has been applied to align the lobe axes with the x and y axes. The shaded curve marks a domain boundary.

2. **"*R* map"** (Supplementary Fig. 13): The tunneling current I is proportional not only to the density of states N integrated within $[0, eV]$, but also a setpoint prefactor $f(x, y, z)$ that involves the tunneling matrix elements, apparent barrier height, and exponential decay of the wave function:

$$I(x, y, z, eV) = f(x, y, z) \int_0^{eV} N(x, y, E) dE. \tag{2}$$

To eliminate setpoint effects, Kohsaka *et al.* (*8*) introduced a ratio map

$$R(x, y, eV) = \frac{I(x,y,z_0,+eV)}{I(x,y,z_0,-eV)} = \frac{\int_0^{eV} N(x,y,E)dE}{\int_{-eV}^{0} N(x,y,E)dE}, \tag{3}$$

which involves acquiring tunneling currents $I(+eV)$ and $I(-eV)$ at the same tip-sample distance z_0, such that the setpoint prefactor is removed by division. We observed x/y-anisotropic lobes using $R(x, y, +300$ mV).

3. **"*Z* map"** (Supplementary Fig. 13): Similarly, the tunneling conductance $g = dI/dV$ also involves the setpoint prefactor:

$$g(x, y, z, eV) = f(x, y, z)N(x, y, eV), \tag{4}$$

which can be removed by computing the ratio (*8*)

$$Z(x, y, eV) = \frac{g(x,y,z_0,+eV)}{g(x,y,z_0,-eV)} = \frac{N(x,y,eV)}{N(x,y,-eV)}. \tag{5}$$

We observed x/y-anisotropic lobes with $Z(x, y, +300$ mV).

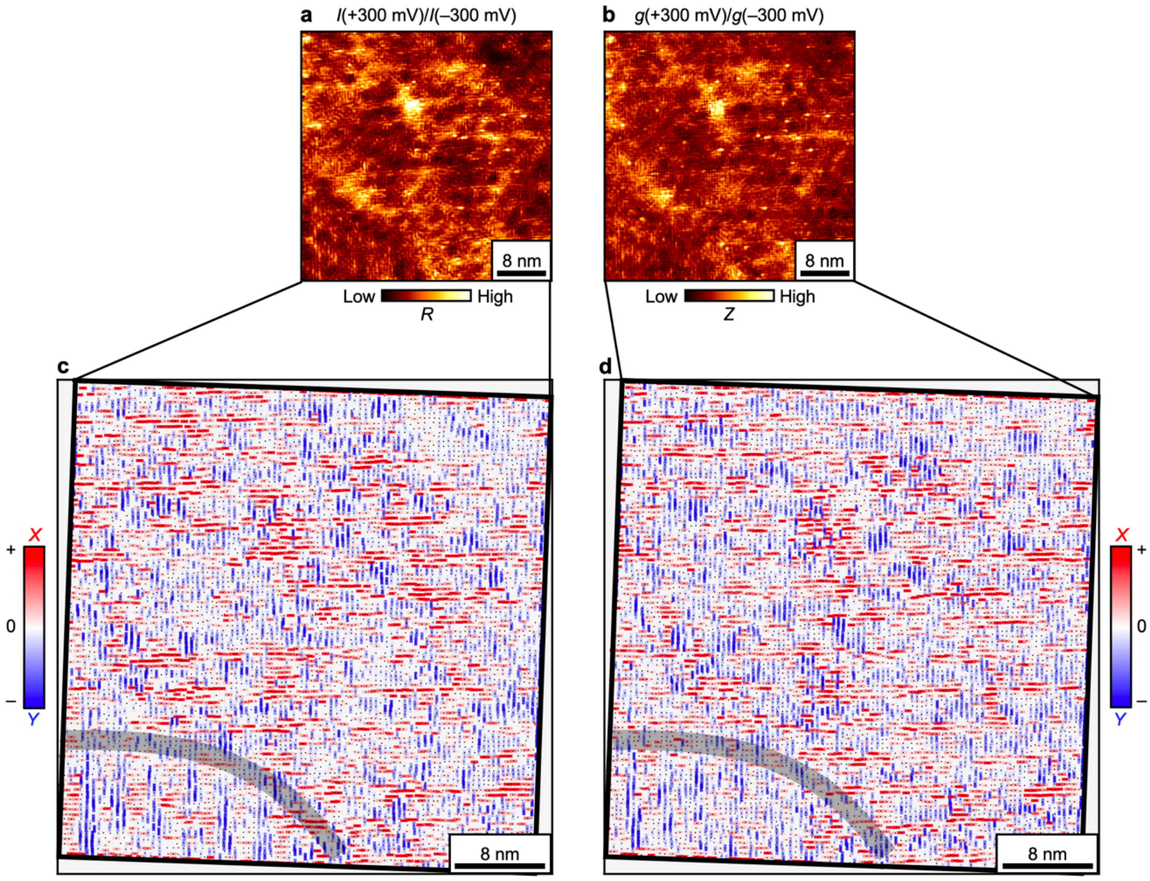

Supplementary Fig. 13: Ratio maps. a, *R* map, defined as the ratio $I(+300$ mV$)/I(-300$ mV$)$. **b**, *Z* map, defined as the ratio $g(+300$ mV$)/g(-300$ mV$)$. **c** and **d**, Maps of lobe anisotropy extracted from **a** and **b**, respectively.

Finally, we discuss the robustness of the histogram analyses shown in Figs. 4f and 5c. We note that Fig. 4 and Supplementary Fig. 8 are +1.0 V topographies taken with different tips (reproduced in Supplementary Figs. 14a and 14b). There are small differences which could be due to tip quality, image resolution, and drift. Nevertheless, the red x-anisotropic lobes consistently spread toward the bottom

half of the CeSbTe unit cell and break the mirror plane intersecting the Te atom along the *x* axis. The blue *y*-anisotropic lobes do not show any definite violation of mirror symmetry.

What we have noticed is that Fourier filtering, while reducing the spatial distribution of the *x* and *y* lobes, does tend to accentuate some additional anisotropies in their spatial distribution, and this has some dependence on the masked FT used (Supplementary Figs. 14c and 14d). For example, with one masked FT, the red *x*-anisotropic lobes appear to shift more toward the left side of the unit cell, but with another masked FT with slightly different choice of CDW satellites, they do not.

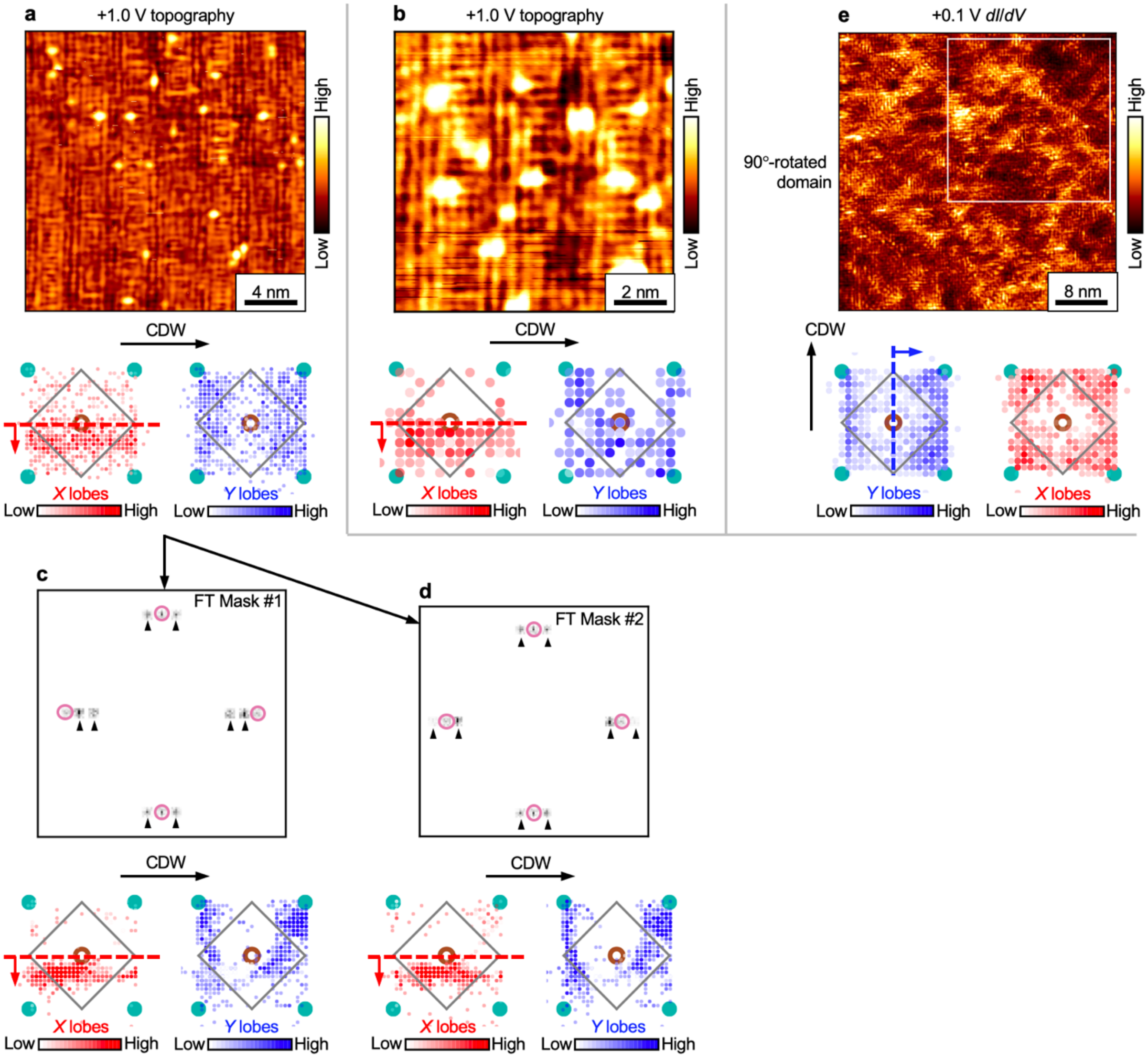

Supplementary Fig. 14. Histogram analysis. a, Histograms of *x*- and *y*-anisotropic lobes derived from the main +1.0 V STM topography in this work (Fig. 3b of main text). The intensity of the red or blue colors is proportional to the number of anisotropic lobes at the given position. The dashed line with solid arrow represents the violation of a mirror plane. **b**, Histograms derived from an additional +1.0 V topography (Supplementary Fig. 8). **c** and **d**, Effects of Fourier filtering of **a** with different masks used. **e**, Histograms derived from a +0.1 V STM *dI*/*dV* map. Only the majority single-domain region with 90°-rotated CDW (white box) is used.

Supplementary Fig. 14e shows a STM *dI*/*dV* map at lower positive biases with a 90°-rotated CDW domain. The anisotropic lobes parallel to the CDW direction (blue in this case) again break a mirror plane intersecting Te, but the positions of these lobes are shifted further away from Te than the previous images. This small shift could be due to the lower bias voltage used, which means there is a differently weighted combination of Sb, Ce and remnant Te states contributing to the tunneling current.

Supplementary Note 7: DFT simulations of STM topographies

Supplementary Fig. 15 shows simulations of STM topographies based on DFT slab calculations of a 1×1 CDW state, where the Sb-Sb bonds show ~8% disproportionation according to the zigzag patterns drawn in Fig. 1f. The simulations cannot reproduce every feature of the more complex 1×7 CDW state, but demonstrates how zigzag Sb $p_{x'}/p_{y'}$ orbitals extend via Ce and remnant Te states to the surface and appear in a simulated STM topography as an elongation of the bright lobes along the *a* direction.

We calculated the integrated local density of states (LDOS) from 0 (=E_F) to +1.0 eV, defined as

$$\rho_{\text{int}}(x, y, z, +1.0\text{ eV}) = \int_{0\text{ eV}}^{+1.0\text{ eV}} N(x, y, z, E)dE, \quad (6)$$

where $N(x, y, z, E)$ is the LDOS derived from the Kohn-Sham orbitals. As ρ_{int} is a function of three variables (x, y, z), one convenient way to visualize this quantity in 3D is to plot a series of iso-amplitude surfaces, $\rho_{\text{int}}(x, y, z, +1.0\text{ eV}) = \rho_0$, for different values of ρ_0. Supplementary Fig. 15 shows four such isosurfaces in sequence of decreasing $\rho_0 = 2.5\times10^{-3}$, 9.5×10^{-4}, 5×10^{-4}, and 2.5×10^{-8} arb. units. The isosurface at $\rho_0 = 2.5\times10^{-3}$ arb. units reveals that the integrated LDOS has highest intensity in the Sb plane and comprises zigzag lobes of the antibonding Sb $p_{x'}/p_{y'}$ orbitals. The isosurfaces at $\rho_0 = 9.5\times10^{-4}$ and 5×10^{-4} arb. units reveal that as the integrated LDOS decreases and spreads toward the surface, it picks up contributions from Ce $dx^2\text{–}y^2$ and remnant Te p_z orbitals. To simulate a constant-current STM topography, which is proportional to an isosurface of constant integrated LDOS, we need to pick ρ_0 small enough, such that the average height of the isosurface lies within the vacuum region of the slab. Supplementary Fig. 15d shows this simulation for $\rho_0 = 2.5\times10^{-8}$ arb. units, which corresponds to an average height ~4.8 Å, which is a typical distance of the tip-sample junction. The simulated +1.0 V topography indeed contains contributions from Te and Ce states, but because the surface Te DOS is largely suppressed in this energy range, fingerprints of the antibonding Sb $p_{x'}/p_{y'}$ orbitals filter through and appear as an anisotropic elongation of the bright lobes (encircled by blue ellipses in Supplementary Figs. 15c–15e) and a redistribution of their weight away from the Te atomic positions. This is consistent with experiments. The anisotropic lobes are visible for other values of ρ_0, e.g., 1×10^{-8} arb. units (Supplementary Fig. 15e).

To further confirm that the anisotropic elongation of the bright lobes originates from subsurface Sb $p_{x'}/p_{y'}$ antibonding orbitals, and not something else, we performed two control calculations:

(1) Simulation of –0.5 V STM topography of the 1×1 CDW (Supplementary Figs. 15a and 15b): The isosurfaces show that the integrated LDOS from 0 to –0.5 eV contains sizable contributions from surface Te p_z orbitals already at $\rho_0 = 2.5\times10^{-3}$ arb. units, which masks any subsurface contributions. The

resulting topography therefore reveals only a square lattice of isotropic bright lobes centered at Te atomic positions, with no signatures of the Sb bond dimers. This is consistent with experiments.

(2) Simulation of +1.0 V STM topography without CDW (Supplementary Figs. 15h and 15i): The isosurfaces show that although the Sb $p_{x'}/p_{y'}$ orbitals contribute significantly to the integrated LDOS, in the absence of CDW, their lobes are isotropic in shape and do not produce any distinctive features in the topography.

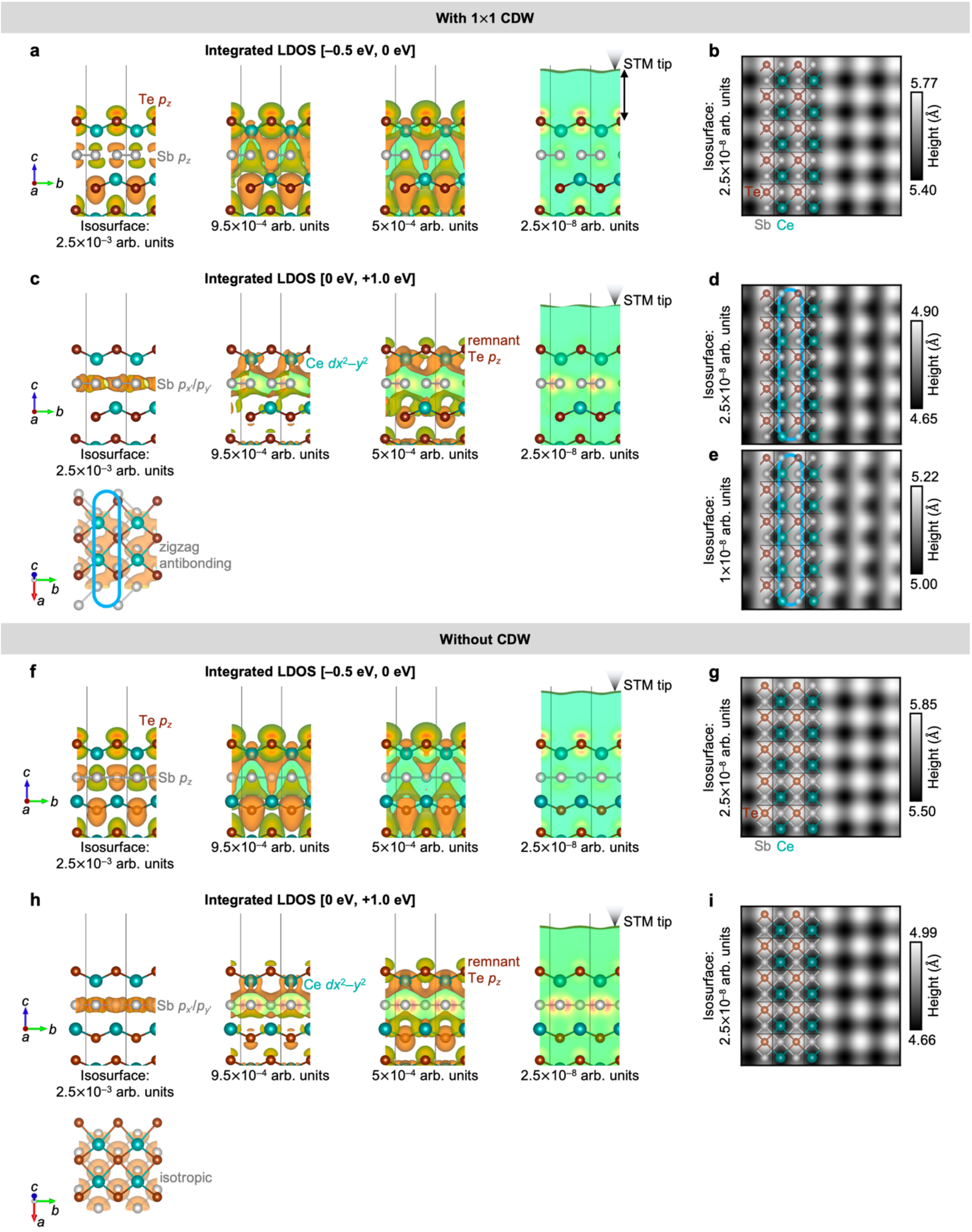

Supplementary Fig. 15: DFT simulations. a–**e**, Results from a CeSbTe slab with 1×1 CDW. **a**, LDOS integrated from −0.5 to 0 eV (=E_F), i.e., $\rho_{int}(x, y, z, -0.5\ \text{eV})$. Four isosurfaces of the same integrated LDOS are shown. **b**, Simulation of −0.5 V STM topography based on the isosurface in **a** at 2.5×10^{-8} arb. units. The height is referenced from the surface Te atom of the slab. **c**, Isosurfaces of the LDOS integrated from 0 to +1.0 eV, i.e., $\rho_{int}(x, y, z, +1.0\ \text{eV})$. Two views of the isosurface at 2.5×10^{-3} arb. units are shown. **d** and **e**, Simulations of +1.0 V STM topographies for two different isosurfaces of the integrated LDOS in **c** at 2.5×10^{-8} and 1×10^{-8} arb. units, respectively. Signatures of the Sb zigzag antibonding orbitals filter through into the simulations (blue ellipses). **f**–**i**, Results from a CeSbTe slab without CDW. **f**, Isosurfaces of the LDOS integrated from −0.5 to 0 eV. **g**, Simulation of −0.5 V STM topography based on the isosurface in **f** at 2.5×10^{-8} arb. units. **h**, Isosurfaces of the LDOS integrated from 0 to +1.0 eV. **i**, Simulation of +1.0 V STM topography based on the isosurface in **h** at 2.5×10^{-8} arb. units.

Supplementary Note 8: Nesting wave vectors and bond-density-wave picture

When the p-electron square lattice with nonsymmorphic symmetry (i.e., two atoms per unit cell) is half filled, the Fermi surface comprises a diamond-shaped pocket with nearly parallel segments (Supplementary Fig. 16a). As explained in the main text, the nesting wave vector of $\mathbf{q} = (2\pi/a, 0)$ results in dimerized chains along x' or y' ($p_{x'}$ and $p_{y'}$ bond density waves) that are phase-shifted relative to each other (Supplementary Fig. 16b).

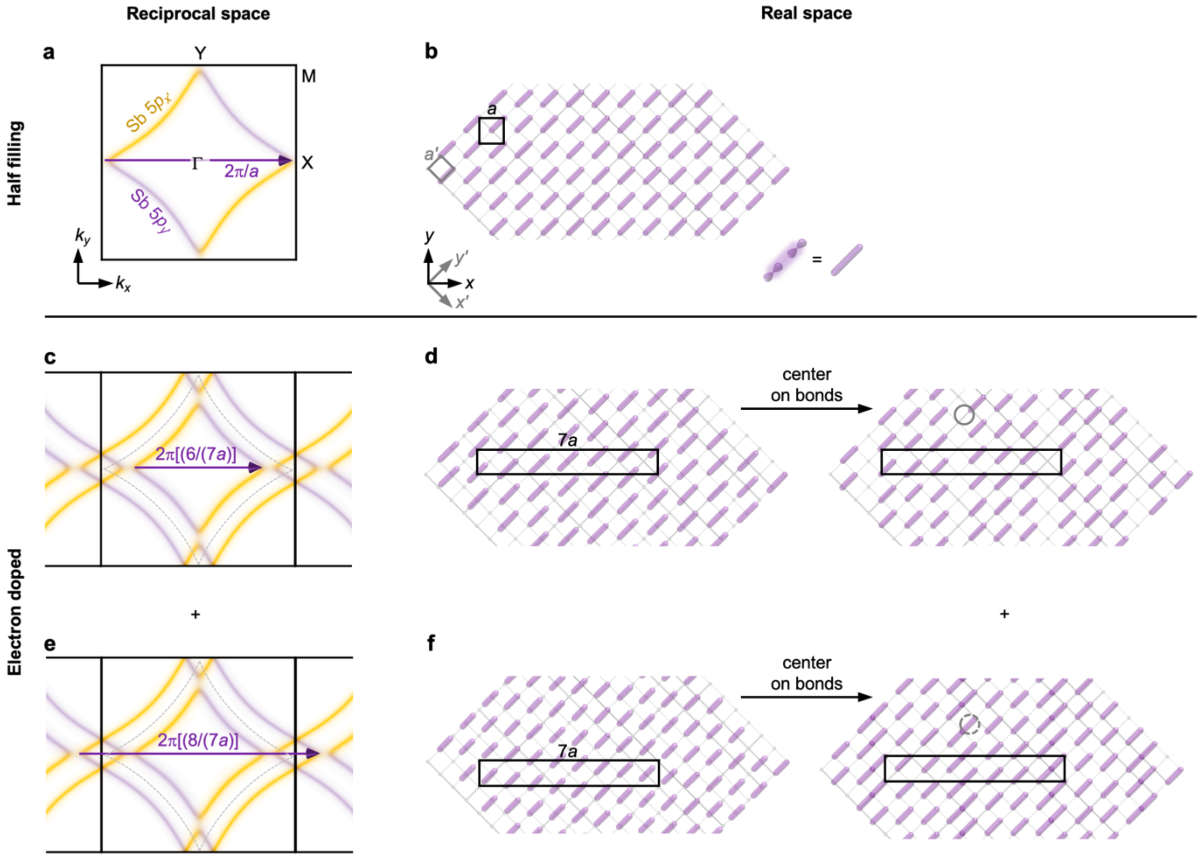

Supplementary Fig. 16: Intrapocket wave vectors and higher harmonics. a, Fermi surface of half-filled p-electron square lattice with nesting wave vector $\mathbf{q} = (2\pi/a, 0)$. **b**, Real-space picture of corresponding bond density wave. For simplicity, only the $p_{y'}$-orbital sector is shown. **c**, Intrapocket wave vector $\mathbf{q} = (2\pi[6/(7a)], 0)$ of the inner Fermi pocket. **d**, The hypothetical bond density wave corresponding to this higher harmonic comprises dimerized chains with periodicity slightly larger than $2a'$, resulting in "missing" dimers (solid circle) when the bonds are properly fixed onto the lattice. **e**, Intrapocket wave vector $\mathbf{q} = (2\pi[8/(7a)], 0)$ of the outer Fermi pocket. **f**, The hypothetical bond density wave corresponding to this higher harmonic comprises dimerized chains with periodicity slightly smaller than $2a'$, resulting in "additional" dimers, i.e., trimers (dashed circle), when the bonds are properly fixed onto the lattice. The actual 1×7 CDW internal structure includes these higher harmonics in **d** and **f** with both kinds of phase slips.

Upon electron doping, the Fermi surface splits into two concentric diamond-shaped pockets. Instead of considering the interpocket wave vector $\mathbf{q} = (2\pi/(7a), 0)$, let us consider the two intrapocket wave vectors. We view the system instead as two sets of pockets, the smaller one with $\mathbf{q} = (2\pi[6/(7a)],$

0) and the larger one with $\mathbf{q} = (2\pi[8/(7a)], 0)$. These intrapocket wave vectors both result in 1×7 CDW, but they highlight distinct higher harmonics involved in the internal structure (Supplementary Figs. 16c–16f). They illustrate how electron doping may be viewed in real space as introducing phase slips into the original 1×1 bond density wave, such that the effective periodicity is enlarged to 1×7.

Supplementary Note 9: Comparison with x-ray diffraction data

Supplementary Fig. 17 presents a comparison of the 1×7 CDW state with the bulk 1×3 and 1×5 CDW states of $Ce(Sb_{1-x}Te_x)Te_{1-\delta}$, which were refined from x-ray diffraction (5). All three CDW patterns comprise zigzag chains of Sb-Sb bonds. We illustrate how each of these CDW patterns can be decomposed into a superposition of $p_{x'}$ and $p_{y'}$ bond density waves. The bond density waves are composed of dimerized chains that (1) are shifted from neighboring chains by a' and (2) contain periodic phase slips. In the 1×3 CDW pattern, the periodic phase slip consists of two consecutive strong bonds, i.e., a trimer. In the 1×5 CDW pattern, the periodic phase slip consists of two consecutive weak bonds. The 1×7 CDW pattern contains both kinds of phase slips.

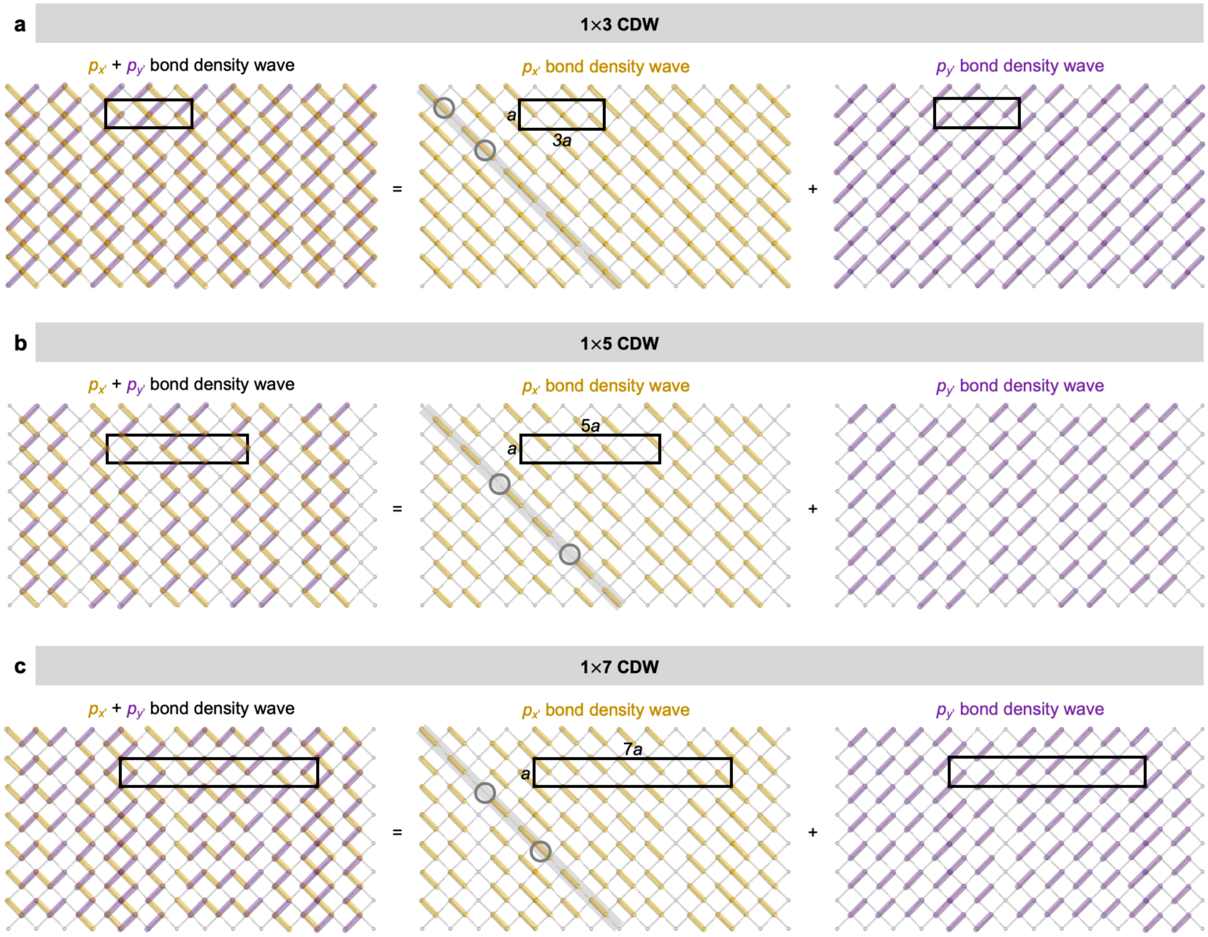

Supplementary Fig. 17: Comparison of CDW internal structures. a and **b**, Decomposition of 1×3 and 1×5 CDW internal structures, as refined by x-ray diffraction (5), into superpositions of Sb $p_{x'}$ and $p_{y'}$ bond density waves. The gray circles mark periodic phase slips along the dimer chains (gray line). **e**, Decomposition of 1×7 CDW internal structure, as determined by STM, into a superposition of Sb $p_{x'}$ and $p_{y'}$ bond density waves. Reproduced from Fig. 6 of main text.

Supplementary Note 10: Multi Pass imaging

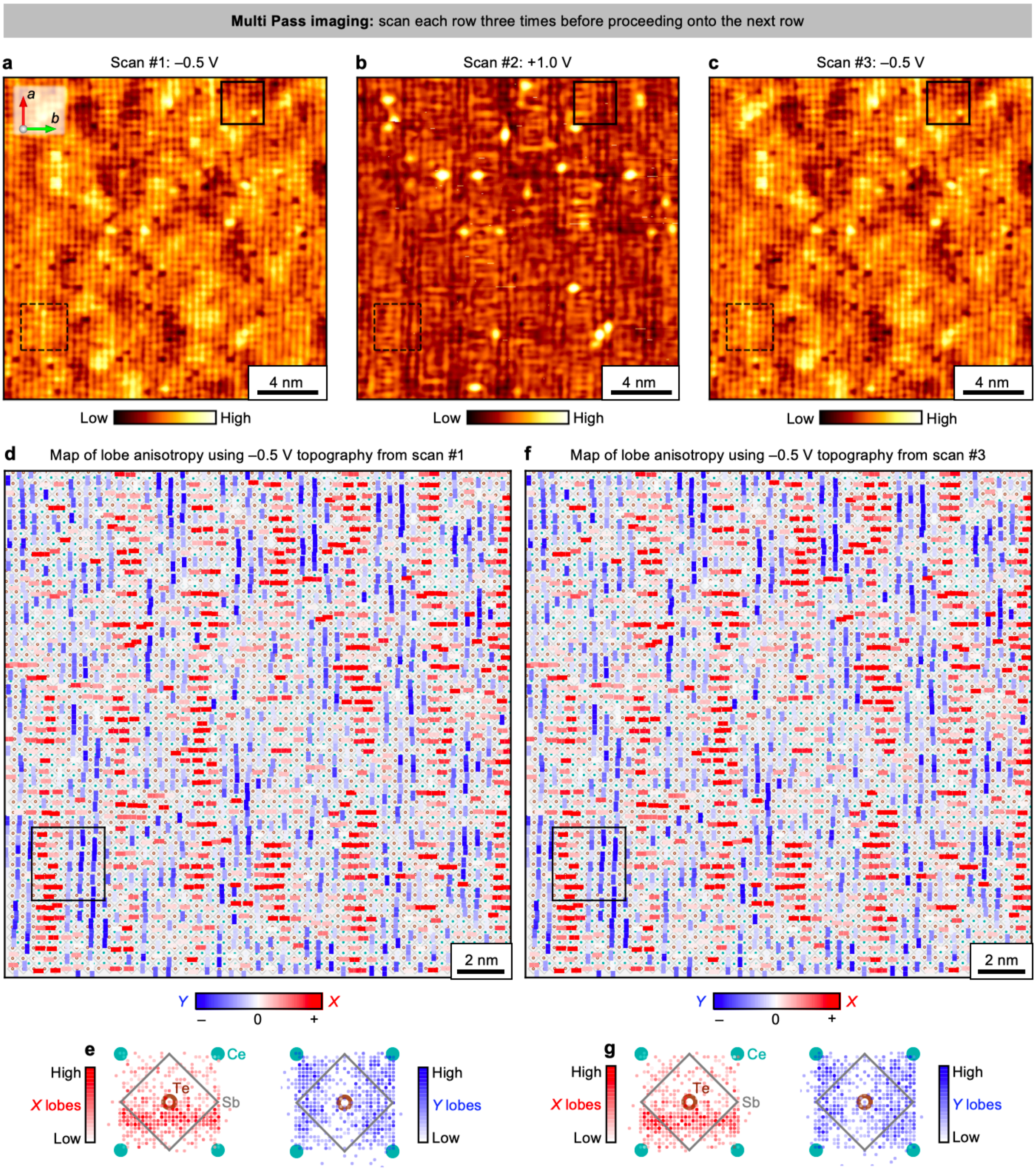

Supplementary Fig. 18: Multi Pass imaging. **a–c**, Simultaneously acquired topographies with biases of –0.5, +1.0, and –0.5 V, respectively. Setpoint current: 20 pA. The topographies in **a** and **b** are reproduced from Figs. 3a and 3b of the main text. **d** and **e**, Map of lobe anisotropy and histogram of lobe distribution generated using the first –0.5 V topography, i.e., scan #1. Reproduced from Figs. 4e and 4f of the main text. **f** and **g**, Map of lobe anisotropy and histogram of lobe distribution generated using the second –0.5 V topography, i.e., scan #3.

To ensure that there was no drift during the acquisition of the –0.5 and +1.0 V topographies in Figs. 3a and 3b of the main text, we utilized the Multi Pass module of the Nanonis SPM Controller software. Over the same field of view, the Multi Pass imaging mode scanned each line three times, with

bias voltages of –0.5, +1.0, and –0.5 V, respectively. Supplementary Fig. 18 shows that the two –0.5 V topographies taken from the first and third scans of each line are nearly identical, and when used as a reference lattice for the analysis of anisotropic lobes, yield nearly identical results.

References

1. S. Klemenz, S. M. Lei, L. M. Schoop, Topological Semimetals in Square-Net Materials. *Annu. Rev. Mater. Res.* **49**, 185 (2019).
2. Y. P. Wang *et al.*, Axial Higgs mode detected by quantum pathway interference in RTe_3. *Nature* **606**, 896-901 (2022).
3. S. M. Lei *et al.*, Charge Density Waves and Magnetism in Topological Semimetal Candidates $GdSb_xTe_{2-x-\delta}$. *Adv. Quantum Technol.* **2**, 1900045 (2019).
4. L. M. Schoop *et al.*, Tunable Weyl and Dirac states in the nonsymmorphic compound CeSbTe. *Sci. Adv.* **4**, eaar2317 (2018).
5. R. Singha *et al.*, Evolving Devil's Staircase Magnetization from Tunable Charge Density Waves in Nonsymmorphic Dirac Semimetals. *Adv. Mater.* **33**, 2103476 (2021).
6. P. Li *et al.*, Charge density wave and weak Kondo effect in a Dirac semimetal CeSbTe. *Sci. China Phys. Mech.* **64**, 237412 (2021).
7. B. Venkatesan *et al.*, Direct Visualization of Disorder Driven Electronic Liquid Crystal Phases in Dirac Nodal Line Semimetal GdSbTe. arXiv:2402.18893v3.
8. Y. Kohsaka *et al.*, An Intrinsic Bond-Centered Electronic Glass with Unidirectional Domains in Underdoped Cuprates. *Science* **315**, 1380 (2007).
